# The Mechanism of Mg^2+^-Mediated Inhibition of Cervical Cancer by Inducing a Senescence-like State via the ATM/CHK2/p21 Signaling Pathway

**DOI:** 10.3390/ijms27104397

**Published:** 2026-05-14

**Authors:** Lei Wang, Yunshan Ouyang, Qian Zhao, Tianshu Wang, Chen Lin

**Affiliations:** 1 Department of Occupational Health and Environmental Hygiene, School of Public Health, Xinjiang Medical University, Urumqi 830000, China; wgi_scout@126.com (L.W.); 19199277613@163.com (Y.O.); 2 Department of Pathology, School of Basic Medical Sciences, Xinjiang Medical University, Urumqi 830000, China; 15966039438@163.com (Q.Z.); wangts0128@163.com (T.W.)

**Keywords:** magnesium ion, cervical cancer, cellular senescence, proliferation, migration

## Abstract

Cervical cancer constitutes a major global health burden with a high incidence rate. Despite its well-established role in genome stability and cell cycle regulation, its specific anti-tumor mechanism involving the induction of a senescence-like state remains unclear. To determine whether Mg^2+^ impedes cervical cancer progression through the induction of a senescence-like phenotype via the ATM/CHK2/p21 pathway, HeLa cells were used in this study. Cell proliferation, migration, and invasion were measured using CCK-8, EdU, wound-healing, and Transwell assays, while SA-β-gal staining and western blotting served to examine both senescence-related markers and pathway protein expression. A BALB/c nude mouse xenograft model was established to evaluate tumor growth and safety following intratumoral Mg^2+^ injection. The results showed that Mg^2+^ inhibited proliferation, migration, and invasion in a concentration-dependent manner. Treatment with 20 mM Mg^2+^ increased SA-β-gal positivity, decreased Lamin B1 expression, and activated the ATM/CHK2/p21 pathway; moreover, this upregulation of p21 was reversed by an ATM inhibitor. ELISA revealed that 10 mM Mg^2+^ enhanced IL-6 and TNF-α secretion, confirming effective induction of the senescence-associated secretory phenotype, while higher concentrations diminished this effect, which may be partly attributed to the reduction in cell viability. In vivo experiments showed that Mg^2+^ inhibited tumor growth without notable alterations in body weight, liver and kidney function, or serum magnesium levels. In summary, the localized high concentration of magnesium ions induces cells to enter a senescence-like state via the ATM/CHK2/p21 pathway, thereby selectively suppressing malignant cellular behaviors. Notably, its in vivo efficacy and safety profile in vivo are favorable. It is also worth noting that these findings should be interpreted within the context of a preclinical, high-dose local Mg^2+^ model.

## 1. Introduction

As a malignant tumor that seriously threatens women’s health, the global epidemiological situation of cervical cancer is worrisome. According to the latest GLOBOCAN 2022 data, there are about 661,000 new cases of cervical cancer in the world every year, and the number of deaths is as high as 348,000, ranking the fourth leading cause of death from female malignant tumors [1]. However, the global burden of cervical cancer is highly uneven, with mortality rates remaining disproportionately high in low- and middle-income countries where organized screening and human papillomavirus (HPV) vaccination programs are not widely implemented. This disparity underscores the urgent need for effective and accessible therapeutic strategies.

As a key mechanism of tumor suppression, cell senescence plays a crucial role in tumor prevention and control. Traditional tumor therapy often involves the direct killing of tumor cells, whereas cell senescence offers a unique tumor-suppression strategy [2]. Senescent cells fundamentally inhibit tumor progression by secreting inflammatory factors (SASPs), stopping proliferation, and eventually being cleared by the immune system [3]. However, tumor cells often evade senescence mechanisms, making the induction of cellular senescence an attractive research direction for tumor therapy [4].

Cervical cancer is uniquely driven by high-risk HPV oncoproteins E6 and E7, which degrade p53 and inactivate Rb, respectively, thereby disrupting DNA damage response (DDR) and cell cycle checkpoints [5,6]. HPV replication inherently activates the DDR to facilitate the viral life cycle, yet HPV-infected cells continue to proliferate despite an active DDR, a feature that renders them heavily dependent on intact DDR pathways for survival [6]. Indeed, the ATM/CHK2 axis is essential for the viability and proliferation of HPV-positive cervical cancer cells, making it an attractive synthetic lethal target [7]. Magnesium ions (Mg^2+^), as essential cofactors for many DDR enzymes [8], may modulate DDR activity and thus interfere with HPV-driven proliferation. By fine-tuning the DDR, Mg^2+^ could potentially push HPV-positive cells from a state of DDR dependency toward growth arrest or a senescence-like state. This provides a specific rationale for investigating Mg^2+^ in cervical cancer, which is distinct from that in other cancer types.

The ATM/CHK2/p21 signaling pathway is a key molecular pathway that regulates cellular senescence [5] and the DNA damage response [9]. As the main kinase of the cellular DNA damage response, the Ataxia Telangiectasia Mutated (ATM) protein can rapidly sense and respond to threats to genomic integrity [10]. When DNA double-strand breaks occur, ATM rapidly phosphorylates and activates the downstream Checkpoint Kinase 2 (CHK2) protein, which in turn leads to the up-regulation of p21 protein expression [11]. p21 acts as a growth suppressor gene, leading to cell cycle arrest in G1 phase and promoting cell senescence or apoptosis [12]. This pathway is not only a key mechanism to maintain genomic stability, but also an important barrier to tumor suppression.

Mg^2+^ is an essential element in the human body, playing an irreplaceable role in maintaining genomic stability, regulating the cell cycle, and DNA damage repair [13]. It has been demonstrated in previous research that Mg^2+^ deficiency promotes DNA damage accumulation through the induction of oxidative stress and pro-inflammatory factor release, which in turn elevates the risk of tumor development [14]. In recent years, the anti-tumor effect of Mg^2+^ has gradually become a research hotspot. Previous studies have confirmed that Mg^2+^ can activate the Caspase-3-dependent apoptotic pathway, inhibit tumor growth [15], and enhance the tumor-killing function of T cells [16]. Previous studies have shown that Mg^2+^ can induce the production of reactive oxygen species (ROS), G0/G1 phase arrest, and cell death in cervical cancer SiHa cells [17]. Based on this, this study further explored another mechanism: whether Mg^2+^ activates the ATM/CHK2/p21 pathway to induce HeLa cells to enter a similar senescent state. It should be emphasized that the Mg^2+^ concentration used in this study (5–20 mmol/L) is much higher than the physiological level and significantly exceeds the normal serum concentration range (0.70–1.10 mmol/L). Therefore, this result is only applicable to the application scenarios of local high-dose Mg^2+^ and can provide a reference for future research on magnesium-based implants or local injection strategies, rather than for nutritional magnesium status or magnesium deficiency. In addition, future studies should include osmotic pressure and anion controls, such as NaCl, to exclude non-specific osmotic pressure effects.

To elucidate the molecular mechanism, this study was designed to determine, based on the above background, if Mg^2+^ can elicit a senescence-like state within cervical cancer cell senescence through the ATM/CHK2/p21 pathway under in vitro and in vivo conditions. The findings may provide a theoretical basis for local Mg^2+^-based interventions in cervical cancer.

## 2. Results

### 2.1. Effect of Mg^2+^ on the Proliferation of HeLa Cells

**Figure 1:**
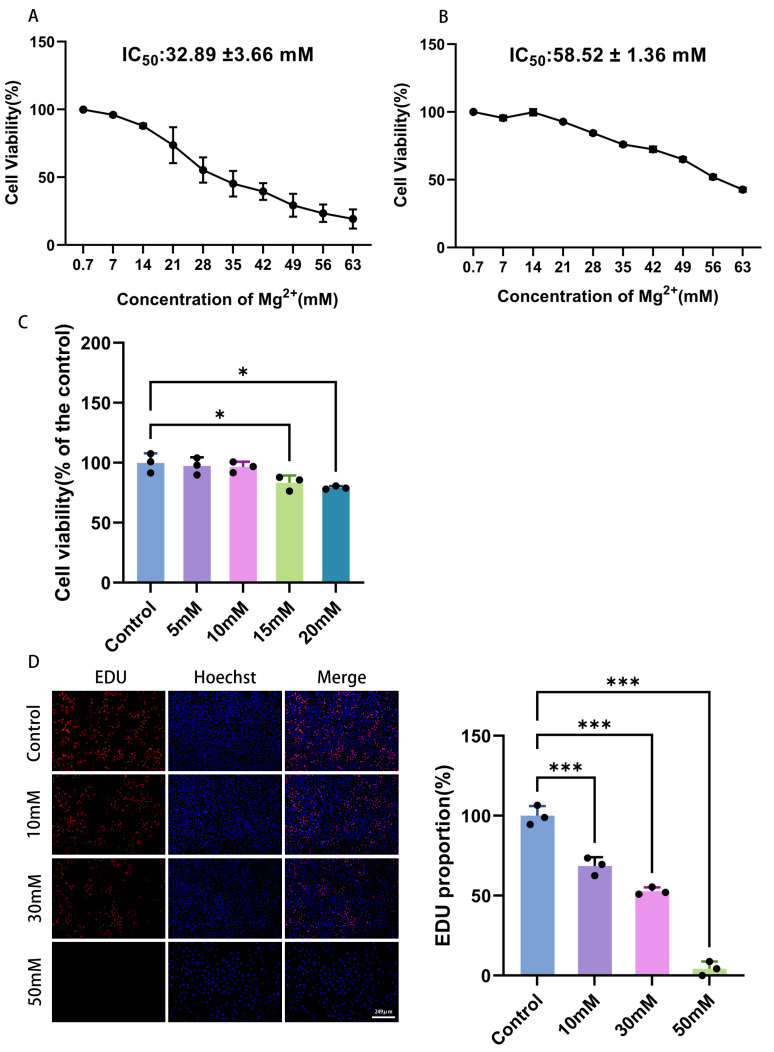
Mg^2+^ inhibits the proliferation of HeLa cells. (**A**) HeLa cell viability after 72 h treatment with Mg^2+^ (0.7–63 mM). IC_50_ = 32.89 ± 3.66 mM. (**B**) H8 cell viability under the same conditions. IC_50_ = 58.52 ± 1.36 mM. (**C**) HeLa cell viability with sub-IC_50_ Mg^2+^ (5–20 mM) by CCK-8. (**D**) EdU staining of HeLa cells at 10, 30, 50 mM MgCl_2_ for 72 h. Data are expressed as mean ± SD compared to the control group; ∗ *p <* 0.05, ∗∗∗ *p <* 0.001.

Different concentrations of Mg^2+^ were applied to HeLa cells to explore their effects on cervical cancer cell viability in vitro, thereby clarifying the role of Mg^2+^ in this context. The reference range for normal serum magnesium levels in humans is 0.70 to 1.10 mmol/L [13]. Therefore, 0.70 mmol/L Mg^2+^ was used as the lower concentration, defined as 1×.The concentration gradient used for the viability assay was 0.7, 7, 14, 21, 28, 35, 42, 49, 56, and 63 mM. As shown in Figure 1A, Mg^2+^ significantly reduced HeLa cell viability in a dose-dependent manner. The IC_50_ of Mg^2+^ for HeLa cells was approximately 32.89 ± 3.66 mM (Figure 1A). Within the same treatment duration, the IC_50_ value of Mg^2+^ for H8 cells was approximately 58.52 ± 1.36 mM, which is significantly higher than that for HeLa cells, suggesting selective cytotoxicity towards cancer cells (Figure 1B). After treating HeLa cells with Mg^2+^ at different concentrations (10 mM, 30 mM, and 50 mM) for 72 h, EdU results showed that the proportion of EdU-positive cells gradually decreased with increasing Mg^2+^ concentration (*p* < 0.001). At 50 mM, EdU-positive cells were scarcely detectable. At 30 mM, the EdU-positive rate was 52.82 ± 2.34%, whereas at 10 mM it was 68.50 ± 5.57% (Figure 1D).

To investigate the potential senescence-inducing effect of Mg^2+^ at concentrations that cause significant growth arrest but avoid massive acute cell death, we selected a sub-IC_50_ concentration gradient (5, 10, 15, 20 mM) for subsequent experiments. As shown in Figure 1C, these concentrations induced varying degrees of growth inhibition while maintaining sufficient cell viability for detecting the senescence phenotype.

### 2.2. Effect of Mg^2+^_2_ on Senescence-like State in HeLa Cells

**Figure 2:**
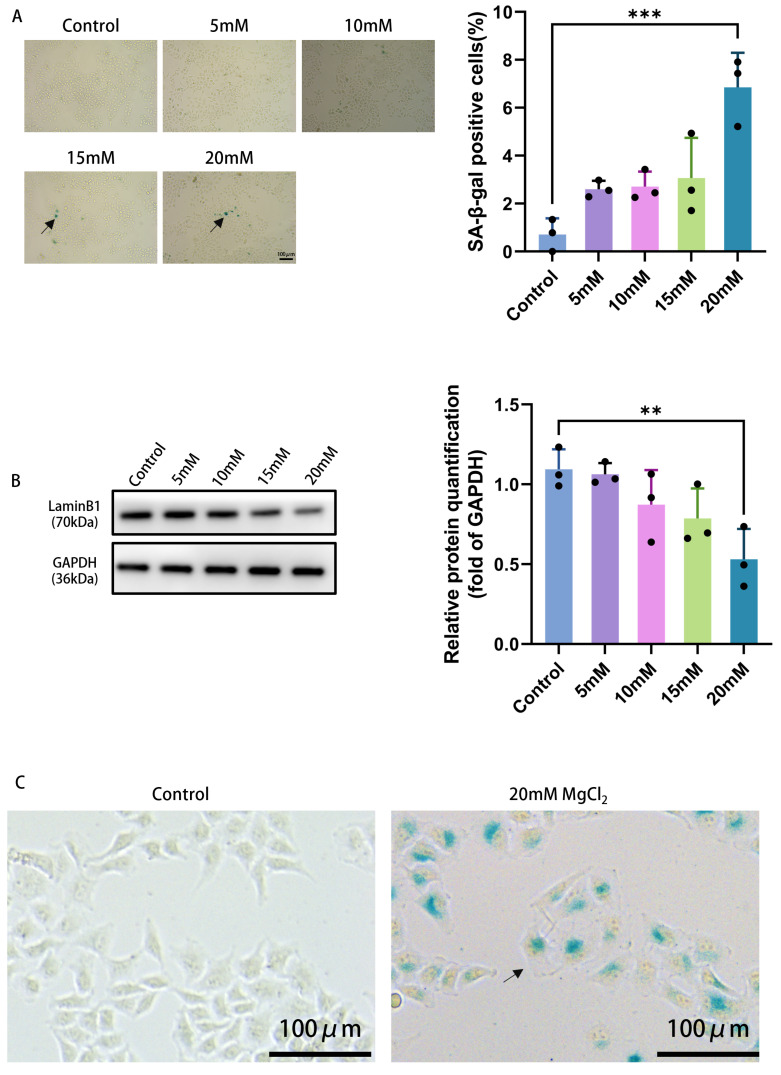
The effect of Mg^2+^ on senescence-like state in HeLa cells. (**A**) SA-β-gal staining of HeLa cells (scale bar: 100 µm; magnification: 100×). Arrows indicate SA-β-gal-positive HeLa cells with senescent morphology. (**B**) effect of Mg^2+^ on LaminB1 protein expression. (**C**) Morphological changes in HeLa cell senescence-like state induced by Mg^2+^, where the magnification factor is 400×. Data are expressed as mean ± SD compared to the control group; ∗∗ *p <* 0.01, ∗∗∗ *p <* 0.001.

Relative to the control group, the percentage of SA-β-gal-positive cells rose in a concentration-dependent fashion across the treatment groups exposed to 5 mM, 10 mM, 15 mM, and 20 mM Mg^2+^. A higher number of SA-β-gal-positive cells was observed in the 20 mM Mg^2+^ group compared to the control group (*p* < 0.001), as illustrated in Figure 2A. This finding implies that Mg^2+^ can induce a similar senescent phenotype. Concurrently, western blot analysis detected a concentration-dependent reduction in relative Lamin B1 protein expression as the Mg^2+^ concentration increased. The difference between the 20 mM Mg^2+^ group and the control group reached statistical significance (*p* < 0.01). Since Lamin B1 downregulation is a key marker of cellular senescence, this result further supports the induction of a senescence-like state by this treatment (Figure 2B).

In comparison to the control condition, the HeLa cells treated with Mg^2+^ exhibited typical morphological characteristics of senescent cells, including flattened cell morphology, vacuolation of the cytoplasm, and significant enlargement of the cell volume. This further indicates that Mg^2+^ can effectively induce a senescence-like state in HeLa cervical cancer cells (Figure 2C).

### 2.3. The Effect of Mg^2+^ on IL-6 and TNF-α in Cervical Cancer Cells

**Figure 3:**
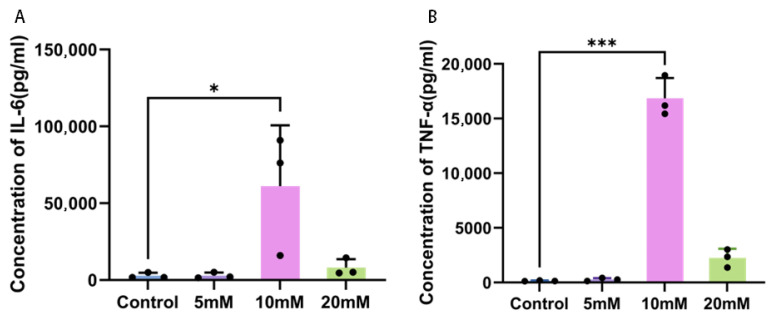
The secretion levels of (**A**) IL-6 and (**B**) TNF-αin the culture supernatants of HeLa cells under different concentration treatments. Data are expressed as mean ± SD compared to the control group; ∗ *p <* 0.05, ∗∗∗ *p <* 0.001.

The results of ELISA indicated that after different Mg^2+^ treatments, there were significant differences in the secretion levels of IL-6 and TNF-α in the supernatant of HeLa cell cultures. No statistically significant differences in interleukin-6 (IL-6) and tumor necrosis factor-α (TNF-α) concentrations were found between the control group and the 5 mM Mg^2+^ group, nor between the 10 mM and 20 mM Mg^2+^ groups (Figure 3A,B). By contrast, the 10 mM Mg^2+^ treatment group exhibited significantly higher IL-6 and TNF-α levels in the cell supernatant than the control, with differences reaching statistical significance (*p* < 0.05, *p* < 0.01). These results suggest that a 10 mM concentration can significantly induce the secretion of IL-6 and TNF-α by HeLa cells. Still, an excessively high concentration may partially inhibit this effect, possibly due to the reduction in cell viability.

### 2.4. Mg^2+^-Induced Alterations in HeLa Cell Motility and Invasiveness

**Figure 4:**
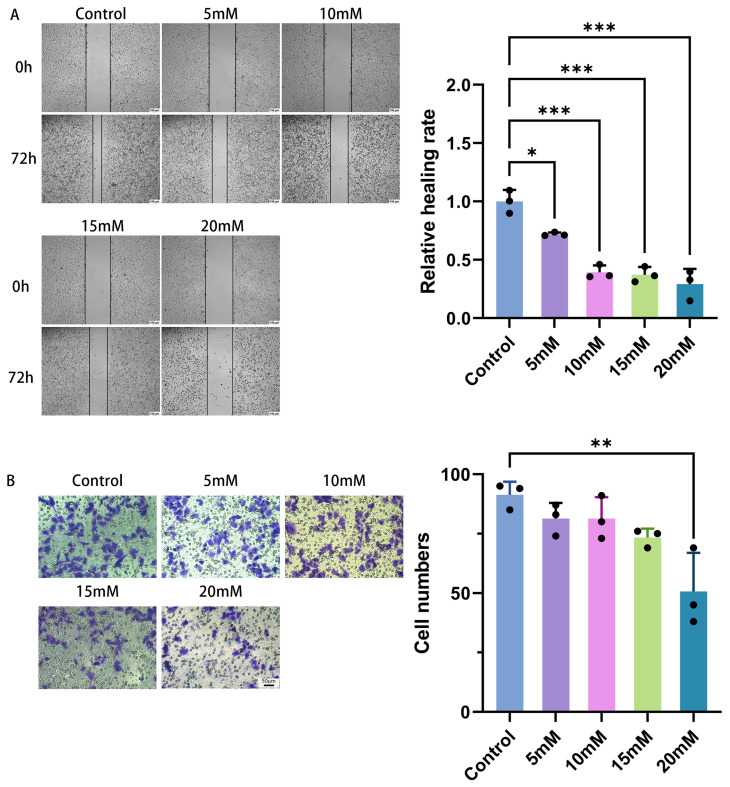
(**A**) Impact of Mg^2+^ on the migratory capability of HeLa cells, with a scale bar of 100 µm; (**B**) effect of Mg^2+^ on the invasive ability of HeLa cells, with a scale bar of 30 µm. Data are expressed as mean ± SD compared to the control group; ∗ *p <* 0.05, ∗∗ *p <* 0.01, ∗∗∗ *p <* 0.001.

Cell senescence represents a stable cell cycle arrest program often accompanied by altered cellular functions. Compared with the control group, HeLa cells treated with different concentrations of Mg^2+^ for 72 h exhibited significantly lower scratch healing rates, as shown in Figure 4A. Higher Mg^2+^ concentrations were associated with reduced cell migration, indicating an inhibitory effect of Mg^2+^ on the migratory potential of HeLa cervical cancer cells. A similar trend was seen for cell invasion: following Mg^2+^ treatment, the number of invasive cells fell significantly in a concentration-dependent manner. This indicates that Mg^2+^ inhibits the invasive capacity of cervical carcinoma HeLa cells, as shown in Figure 4B. Both scratch repair and Transwell assay results demonstrate that cell migration and invasive abilities significantly diminish with increasing Mg^2+^ concentration, consistent with the proliferation-inhibitory phenotype.

### 2.5. Effect of Mg^2+^ on ATM/CHK2/p21 Signaling Pathway in HeLa Cells

**Figure 5:**
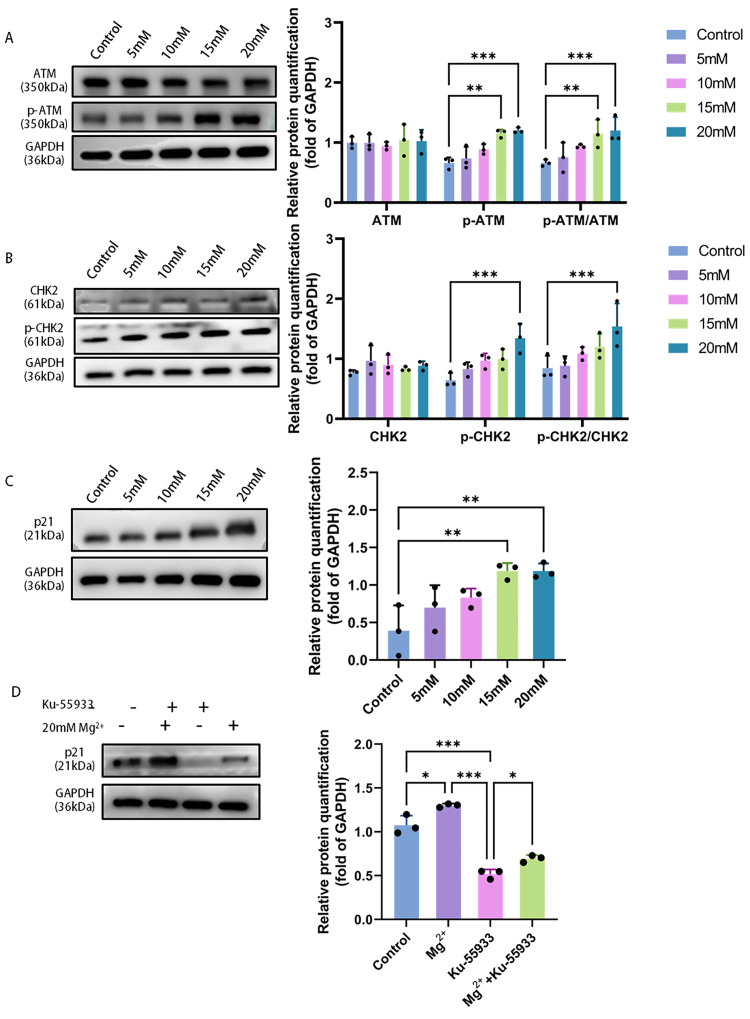
Protein expression levels in HeLa cells following treatment with varying Mg^2+^ concentrations were analyzed by western blotting: (**A**) ATM and p-ATM; (**B**) CHK2 and p-CHK; (**C**) p21; (**D**) p21 expression in the 20 mM Mg^2+^ group with or without KU-55933 (ATM inhibitor) co-treatment. Data are expressed as mean ± SD compared to the control group; ∗ *p <* 0.05, ∗∗ *p <* 0.01, ∗∗∗ *p <* 0.001.

In HeLa cells treated with 20 mM Mg^2+^, no significant change was observed in the expression of ATM and CHK2 proteins, whereas their phosphorylation levels were significantly upregulated (*p* < 0.001). Additionally, p21 protein expression was also increased (*p* < 0.01), as shown in Figure 5A,B. Following treatment with the specific ATM inhibitor KU-55933, the upregulation of p21 expression was significantly suppressed compared to the 20 mM Mg^2+^ treatment group (*p <* 0.001), suggesting that ATM activation is required for Mg^2+^-induced p21 upregulation.

### 2.6. Mg^2+^ Suppresses Tumor Growth In Vivo

**Figure 6:**
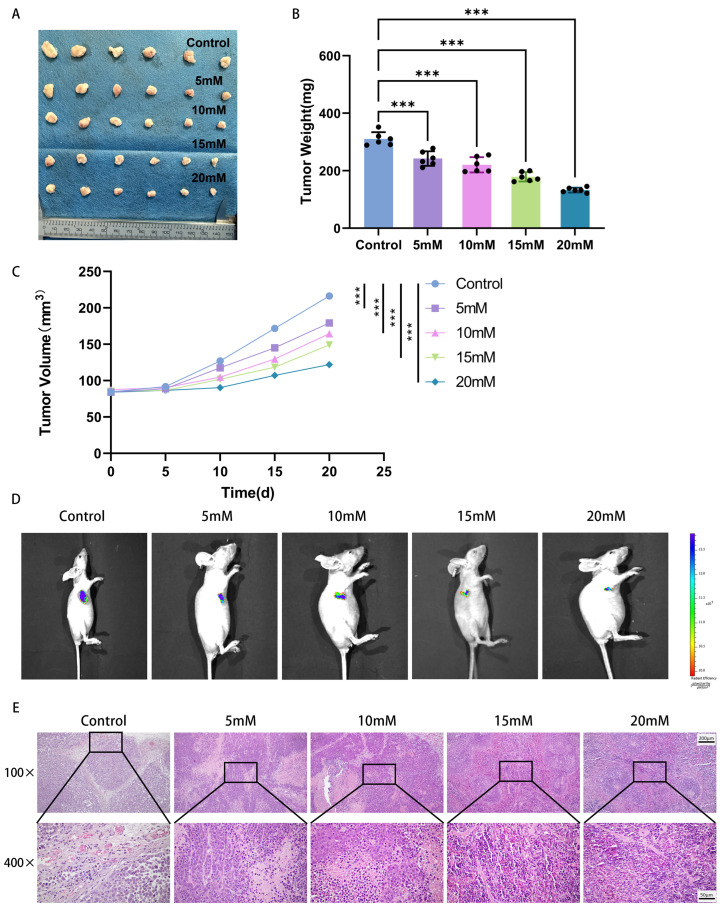
(**A**) Xenograft tumor images (day 20, *n* = 6), (**B**) tumor mass, (**C**) tumor volume alterations, (**D**) bioluminescence imaging of nude mouse tumors after injection with distinct Mg^2+^ concentrations, and (**E**) H&E-stained tumor histology; scale bar length = 200 μm (100×) and 50 μm (400×). Data are expressed as mean ± SD compared to the control group; ∗∗∗ *p <* 0.001.

To further evaluate the therapeutic potential of Mg^2+^ against cervical cancer, a subcutaneous xenograft mouse model was established using GFP-labeled HeLa cells. Twenty days after intratumoral Mg^2+^ injection, the tumors from each group are presented in Figure 6A. As shown in Figure 6B,C, both the average mass and volume of tumors in the Mg^2+^-treated group were significantly lower than those in the control group (*p* < 0.05). In vivo imaging analysis of small animals further revealed that intratumoral Mg^2+^ injection reduced tumor size relative to the control group, with few metastases observed in the surrounding organs (Figure 6D). H&E staining demonstrated that cells in the control group and the 5 mM Mg^2+^ treatment group retained intact morphology, regular arrangement, normal nuclear-to-cytoplasmic ratios, and no obvious age-related morphological alterations. In the 10 mM Mg^2+^ group, the cell tissue was relatively dense at low power (×100), and some cells began to show early senescence-like characteristics such as karyopyknosis and cytoplasmic vacuolization at high power (×400). There were also nuclear pleomorphism and nucleolar blurring. In the 15 mM Mg^2+^-treated group, the tissue showed a uniform purple staining area at low power, and a large number of cells showed typical morphological characteristics of a senescence-like state at high power. These changes include nuclear hyperchromasia, karyopyknosis, cell shrinkage, and loss of intercellular connections. In the 20 mM Mg^2+^-treated group, the tissue was dense at low magnification, and the cells showed significant senescence-like characteristics in almost the whole field of view at high magnification. Abnormal nucleocytoplasmic ratio, severe nuclear atypia, and marked cellular structural damage were the main clinical manifestations. These results indicate that intratumoral injection of high concentrations of Mg^2+^ can significantly induce a senescence-like state in cervical cancer cells, and with the increase in Mg^2+^ concentration, the degree of senescence-like changes in cells is dose-dependent, and the histopathological changes are more significant (Figure 6E).

### 2.7. Biosafety Assessment

**Figure 7:**
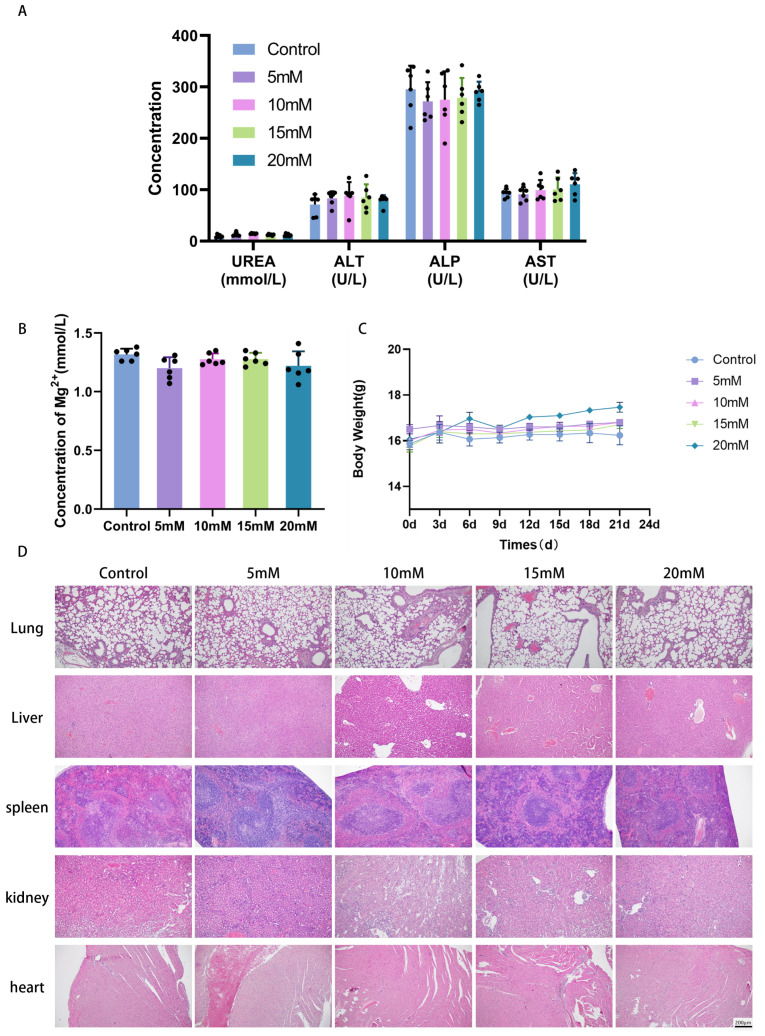
Mice in each group were injected with MgCl2 for 20 days. (**A**) Blood levels of alanine aminotransferase (ALT), aspartate aminotransferase (AST), alkaline phosphatase (ALP), and UREA; (**B**) blood magnesium concentration; (**C**) changes in body weight of mice in each group; and (**D**) H&E stained images of major organs, with a scale bar of 200 μm. Data are expressed as mean ± SD compared to the control group.

All mice exhibited normal locomotion, foraging, and drinking after Mg^2+^ treatment. No significant differences in blood biochemical markers (ALT, AST, ALP, urea) were detected among groups (Figure 7A; *p* > 0.05), reflecting normal hepatic and renal function following Mg^2+^ injection. Intergroup comparisons also showed no significant differences in serum magnesium concentration (Figure 7B; *p* > 0.05) or body weight (Figure 7C; *p* > 0.05). H&E staining of major organs revealed no histological changes in Mg^2+^-treated mice compared to controls (Figure 7D). These results demonstrate that intratumoral injection of an appropriate Mg^2+^ concentration offers a degree of biological safety.

## 3. Discussion

Ranking as the fourth most abundant cation in the human body, Mg^2+^ is crucial for key physiological functions such as safeguarding genomic stability and modulating cell proliferation and apoptosis [18]. A close relationship between Mg^2+^ and tumor development has been demonstrated in recent studies: Mg^2+^ deficiency is linked to an elevated risk of diverse tumors [19], while the addition of Mg^2+^ can hinder tumor growth [20]. However, the specific anti-tumor mechanism of Mg^2+^, especially in cervical cancer, remains to be elucidated. This study systematically revealed that Mg^2+^ could act as a potential anti-tumor factor by specifically activating the ATM/CHK2/p21 signaling pathway, inducing a senescence-like state, and effectively inhibiting the proliferation, migration, and invasion of cervical cancer HeLa cells, and providing new molecular biological evidence for further understanding the anti-tumor effect of Mg^2+^. It could also lay an important theoretical foundation for the development of novel Mg^2+^-based anti-tumor treatment strategies.

### 3.1. Effect of Mg^2+^ on the Malignant Biological Behavior of Cervical Cancer

By regulating the activity of multiple enzymes, Mg^2+^—an essential second messenger in the body—takes part in a broad spectrum of vital biological processes, such as DNA repair, cell cycle regulation, and energy metabolism [21]. A concentration-dependent inhibition of HeLa cell proliferation, invasion, and migration was observed upon Mg^2+^ treatment, as evaluated by CCK-8, EDU, wound healing, and Transwell invasion assays. For various types of cancer, magnesium ions have similar anti-tumor effects, although the underlying mechanisms may differ. In osteosarcoma, magnesium and magnesium-6 silver can reduce cell migration, invasion, and angiogenesis caused by cancer [22]. Mg^2+^ derived from degraded high-purity magnesium wires markedly inhibits ovarian tumor growth and increases apoptosis [20]. Likewise, Mg^2+^ suppresses gallbladder cancer cell growth and induces apoptotic death [23]. In colorectal cancer, Mg^2+^ acts through a distinct but related mechanism, causing cell cycle arrest and caspase-3-dependent apoptosis [15]. It is worth noting that previous studies on cervical cancer SiHa cells have shown that Mg^2+^ can induce G0/G1 phase arrest and cell apoptosis [17]. However, this study revealed a unique mechanism in HeLa cells: Mg^2+^ induces cells to enter a similar senescent state through the ATM/CHK2/p21 pathway, rather than mainly triggering cell apoptosis. Taken together, these findings indicate that Mg^2+^ exhibits anti-tumor activity across multiple cancer types, although the specific mechanisms—ranging from apoptosis and cell cycle arrest to senescence-like induction—may vary depending on the tumor type and cellular context.

Notably, within the same concentration range, Mg^2+^ had no significant effect on the cell viability of normal cervical epithelial H8 cells, suggesting that Mg^2+^ may have an ideal therapeutic window, which can effectively inhibit tumor cells with relatively little damage to the surrounding normal tissues, suggesting its potential application in tumor-targeted therapy. Quantitatively, the IC_50_ of Mg^2+^ for HeLa cells was 32.89 ± 3.66 mM, whereas that for H8 cells was significantly higher at 58.52 ± 1.36 mM, indicating that H8 cells are approximately 1.8-fold more resistant to Mg^2+^-induced growth inhibition. This selectivity may stem from fundamental differences in physiological properties between tumor cells and normal cells. First of all, tumor cells are usually in a state of high proliferation and metabolic stress [24], and their ion homeostasis regulatory system may be more vulnerable and sensitive to drastic changes in ion concentration in the external environment [25]. Secondly, the expression and function of Mg^2+^ transporters in tumor cells may be abnormal, resulting in a different response pattern to extracellular Mg^2+^ concentration compared with normal cells [26,27]. Recent evidence indicates that alterations in the expression and activity of magnesium transporters, including MAGT1, TRPM6/7, and CNNM proteins, are frequently observed in cancer cells and human tumor tissues. These transporters regulate multiple cancer cell hallmarks and oncogenic signaling pathways [28,29]. Third, in addition to transporter abnormalities, mitochondria could act as a critical connection allowing Mg^2+^ to regulate tumor cell behavior. By controlling energy metabolism and oxidative stress levels, Mg^2+^ differentially impacts the proliferation and invasion of cancer cells when compared with normal cells [30]. Fourth, at a systemic level, magnesium homeostasis scores (MHS) reflect the integrated status of magnesium-related gene expression. These scores are reduced in cancers and correlate with lower tumor mutational burden, microsatellite instability, and immune dysfunction [31]. The distinct MHS profiles between HeLa and H8 cells may further contribute to their differential sensitivity to Mg^2+^. Collectively, these factors, including metabolic vulnerability, altered Mg^2+^ transporter expression, mitochondrial regulation, and global magnesium homeostasis signatures, provide a multi-level explanation for why HeLa cells exhibit a higher degree of susceptibility to the inhibitory effect of Mg^2+^ on growth relative to normal H8 cells. However, this study only utilized one HPV-positive cervical cancer cell line (HeLa) and one immortalized normal cervical epithelial cell line (H8), which limits the general applicability of the results of this study. Future research should include more HPV-positive cell lines, such as SiHa and CaSki, as well as HPV-negative cell lines, such as C33A, to verify the similar aging effects observed under different genetic backgrounds.

### 3.2. Mg^2+^ Induced Cell Senescence by Activating the ATM/CHK2/p21 Pathway

A crucial protective mechanism, cellular senescence allows the body to hinder tumor emergence and progression [32]. Cellularly, senescence is marked by sustained cell-cycle arrest, which efficiently blocks the limitless proliferation of tumor cells and constitutes a key endogenous defense system against tumorigenesis [4]. This study investigated whether Mg^2+^ induces a senescence-like state in HeLa cells. Treatment with 20 mM Mg^2+^ for 72 h, a concentration below the calculated IC_50_ (32.89 ± 3.66 mM), led to a significant senescence-like phenotype. Specifically, a significant rise was observed in the proportion of SA-β-gal-positive cells, a common indicator of cellular senescence, accompanied by downregulation of Lamin B1, which serves as a negative senescence marker. These observations, together with the concentration-dependent growth arrest, support the induction of a senescence-like state by Mg^2+^. Thus, Mg^2+^ can effectively drive cervical cancer cells toward a senescence-like phenotype, offering a potential avenue for senescence-based cancer therapy. Nevertheless, it is acknowledged that distinguishing early senescence from pre-apoptotic stress under these conditions requires further investigation, including long-term clonogenic assays, Annexin V/PI staining to exclude apoptosis, and detection of DNA damage markers such as γH2AX. These limitations need to be addressed in future studies.

Based on the confirmation that Mg^2+^ can effectively induce cervical cancer cells to enter a similar senescent state, this study further investigated whether different concentrations of magnesium ions could regulate the key inflammatory factors in the senescence-associated secretory phenotype (SASP) in HeLa cells. As demonstrated by this study, a 10 mM Mg^2+^ concentration significantly stimulated IL-6 and TNF-α secretion from senescent-like cervical cancer cells, while 5 mM and 20 mM had no significant effect. This indicates that the induction effect is concentration-dependent and there is a “peak effect”. We acknowledge that only IL-6 and TNF-α were measured, which is insufficient to fully describe the SASP; future research should examine more extensive SASP factors, such as IL-8 and matrix metalloproteinases. The senescence-associated secretory phenotype (SASP) is an important characteristic of senescent cells, and its secretion pattern is finely regulated by multiple signaling pathways. The 10 millimole concentration may be precisely within the threshold range for activating key pathways effectively. The transcription process of inflammatory factors is initiated through the DNA damage response (DDR) or the cGAS-STING pathway [33]. At 20 mM, an excessive level of stimulation might conversely set off negative feedback regulation, for instance by promoting the production of anti-inflammatory cytokines or driving cells into a heightened stress state, consequently blocking the release of IL-6 and TNF-α [34]. Furthermore, the decreased levels of cytokines at a 20 millimolar concentration may partly reflect the decline in cell viability rather than merely being a purely biological regulatory effect, because the high concentration environment can damage the overall cell state. Such a non-proportional dose-response correlation has been detected in studies assessing how pharmacological agents exert control over the tumor microenvironment [35]. Furthermore, the differential regulation of SASP at different concentrations may reflect the adaptation mechanisms of tumor cells when responding to different intensities of stress, that is, by dynamically adjusting the secretion pattern to gain survival advantages. This investigation furnishes experimental data for devising anti-neoplastic strategies based on the manipulation of SASP.

At the same time, this study confirmed that Mg^2+^ exerts a pro-aging effect by activating the ATM/CHK2/p21 signaling pathway, which could be attributable to the activation of the DNA damage response (DDR) [36]. Mg^2+^ deficiency has been shown to impair DNA stability, whereas adequate Mg^2+^ is necessary to maintain genome integrity as it is an essential cofactor for a variety of DNA repair enzymes, such as those in NER, BER, and MMR systems [37,38]. In HeLa cells, the total protein levels of ATM and CHK2 showed no significant response to Mg^2+^ treatment, as determined by western blot; however, the phosphorylated forms p-ATM and p-CHK2 were significantly upregulated, demonstrating that Mg^2+^ activates the canonical ATM/CHK2 axis. ATM is the core kinase of DNA double-strand breaks (DSBs), and its autophosphorylation at the damage site is a key step in initiating the entire DDR signaling network [39]. Activated ATM then phosphorylates its downstream key substrate CHK2, forming a cascade amplification effect to transmit the damage signal [40]. While the exact upstream trigger for ATM activation by high-concentration Mg^2+^ remains to be elucidated, our data clearly place ATM activation as a central event.

In the DDR pathway, p21 is a key effector protein that responds to ATM/CHK2 signaling and performs cell cycle arrest [41]. As a broad-spectrum cyclin-dependent kinase (CDK) inhibitor, its upregulation can effectively inhibit the activities of CDK2 and CDK4/6, which arrests the cell cycle at G1/S or G2/M—a key event marking the onset of cellular senescence [42]. This study revealed that p21 protein expression rose as Mg^2+^ concentration increased, a pattern that aligned completely with the observed phenotypes of suppressed proliferation and induced senescence and directly revealed the molecular bridge connecting the upstream signal and downstream effect. Despite the absence of direct flow cytometric measurement of cell cycle distribution in this study, the concentration-dependent upregulation of p21 (which is a key regulator of cell cycle arrest) and the increase in SA-β-gal positive rate and the decrease in Lamin B1 expression support the occurrence of cell cycle arrest and similar aging phenotypes. Admittedly, this constitutes a limitation of the current study and will be remedied in future research.

To definitively establish the causal relationship in this signaling pathway, we employed KU-55933, a specific ATM inhibitor, for intervention experiments. The results demonstrated that the upregulation of p21 protein induced by high concentrations of Mg^2+^ was significantly reversed following KU-55933 treatment. These rescue assay results confirmed that ATM kinase activation served as an essential upstream event for p21 upregulation.

### 3.3. The Antitumor Effect and Safety of Mg^2+^ in Nude Mice Were Evaluated

In the current investigation, the efficacy and safety of Mg^2+^ against tumors were systematically assessed through a xenograft model of cervical cancer in BALB/c nude mice. Administration of Mg^2+^ directly into the tumor site produced a marked reduction in cervical cancer growth. Histopathological examination revealed that tumor tissues from the Mg^2+^-treated group exhibited characteristic senescence-associated alterations, such as enlarged cell size and a reduced nuclear-to-cytoplasmic ratio, while no significant inflammatory cell infiltration or tissue necrosis was detected. These results suggested that Mg^2+^ exerted anti-tumor effects probably through inducing a senescence-like state of tumor cells rather than cytotoxicity. A key consideration for translating these findings is the local concentration achieved within the tumor after injection. While our study demonstrates efficacy with injected concentrations of 10–20 mM, the actual sustained intratumoral Mg^2+^ concentration remains undefined. Intratumoral injection of Mg^2+^ did not cause significant weight loss or abnormal liver and kidney function indicators in mice, suggesting that it had good biosafety. However, the histological evidence of tissue aging in this study was not fully supported by immunohistochemical techniques. Future research should include immunostaining for p21 and Ki-67, and ideally also for γH2AX, to strengthen this conclusion. Additionally, this study did not investigate the pharmacokinetics (including absorption, distribution, and local concentration) of the injected magnesium ions within the tumors. These limitations should be addressed in subsequent studies.

As an endogenous substance, Mg^2+^ has the advantages of low toxicity, good biocompatibility, wide sources, and low cost. It is expected to be developed as a new adjuvant therapeutic agent for cancer, especially for patients who tolerate traditional chemotherapy or require long-term maintenance treatment. However, there are still some limitations in this study: (1) the different effects of Mg^2+^ on cervical cancer cells with different genotypes (such as HPV positive/negative) have not been systematically evaluated; in particular, only one HPV-positive line (HeLa) and one immortalized normal line (H8) were used; future studies should include HPV-negative lines (C33A) and additional HPV- positive lines (SiHa, CaSki); (2) whether long-term Mg^2+^ treatment induces adaptive drug resistance of tumor cells or not is unclear; (3) the definitive distinction between a senescence-like state and a pre-death stress response at the effective concentrations warrants further investigation using assays for long-term proliferation arrest (e.g., clonogenic assay), apoptosis exclusion (Annexin V/PI), and direct DNA damage markers (γH2AX, 53BP1); (4) the precise intratumoral pharmacokinetics of injected Mg^2+^ and the upstream mechanism triggering ATM activation require further exploration; and (5) the optimal dose, route, and duration of Mg^2+^ administration need to be further optimized by pharmacodynamic and pharmacokinetic (PD-PK) studies. In the future, single-cell transcriptome sequencing (scRNA-seq) and spatial transcriptome technology can be combined to further analyze the regulatory effects of Mg^2+^ on the tumor microenvironment (such as immune cell infiltration and matrix remodeling). At the same time, the combined treatment strategy of Mg^2+^ with radiotherapy, immune checkpoint inhibitors, or targeted drugs will be explored to provide a new theoretical basis and clinical transformation direction for the precise and individualized treatment of cervical cancer.

## 4. Materials and Methods

### 4.1. Cell Culture

Human cervical adenocarcinoma cells (HeLa, QS-H085) and immortalized human normal cervical epithelial cells (H8, QS-H283) were purchased from Qisai Biotechnology Co., Ltd. (Wuhan, China). Cells were cultured in DMEM (Gibco, Grand Island, NY, USA) supplemented with 10% fetal bovine serum (FBS, Sigma, Darmstadt, Germany) and 1% penicillin-streptomycin (Gibco, USA). All cells were maintained in a 37 °C incubator with 5% CO_2_. The mycoplasma test and STR identification were conducted on the cell lines used.

### 4.2. CCK-8 Assay

Dispense HeLa and H8 cells into 96-well plates at 5 × 10^3^ cells/well. After 24 h of culture, replace the medium with complete medium containing different concentrations of Mg^2+^. Prepare the CCK-8 working solution by mixing Cell Counting Kit-8 (CCK-8, Proteintech, Chicago, IL, USA) with DMEM at a 1:10 ratio. After 72 h of culture, remove the old medium and add 100 μL of CCK-8 working solution to each well. Incubate the plate in a culture incubator for 1 h, then measure the absorbance of each well using a microplate reader (Multiskan FC, Thermo Scientific, Waltham, MA, USA). Calculate the IC50 as the Mg^2+^ concentration required to inhibit 50% of cell growth. The concentrations of magnesium chloride were 0.7, 7, 14, 21, 28, 35, 42, 49, 56, and 63 mM, and the treatment duration was 72 h. IC_50_ values were calculated using GraphPad Prism 9.0 software with nonlinear regression (log(inhibitor) vs. normalized response-variable slope).

### 4.3. EdU Assay

After seeding logarithmic-phase HeLa cells into 6-well plates (1.5 × 10^5^ cells/well), the control group received complete medium, and the treatment groups received complete medium containing Mg^2+^ at 10, 30, and 50 mmol/L, respectively. EdU cell proliferation assay kit (Beyotime, Shanghai, China) was used according to the instructions: following fixation in 4% paraformaldehyde and permeabilization in 0.5% Triton X-100, the cells were incubated for 30 min with Click reaction solution in the dark and then stained with DAPI. The images were observed and acquired under a fluorescence inverted microscope (DMi8 automated, Leica, Wetzlar, Germany).

### 4.4. SA-β-Gal Staining Analysis

Log-phase HeLa cells were seeded in 6-well plates at a density of 1.5 × 10^5^ cells/well. The experimental groups were treated with complete medium containing different concentrations of Mg^2+^ for 72 h, whereas the control group was cultured in unsupplemented complete medium. At the predetermined time point, the culture medium was discarded, and the cells were washed twice with PBS. The cells were fixed with β-galactosidase staining fixative (4% paraformaldehyde) at room temperature for 15 min, then washed three times with PBS for 5 min each. The cells were then supplemented with freshly prepared SA-β-gal staining working solution (Senescent Cell β-Galactosidase Staining Kit, Servicebio, Wuhan, China) and incubated for 12 h at 37 °C in the dark. Upon completion of staining, the cells were subjected to two PBS washes to cease the reaction and thereafter sealed with glycerol. Cell morphology changes were observed under a fluorescence inverted microscope (DMi8 automated, Leica, Germany) in bright field mode, and images were captured at magnifications of 100× and 400×.

### 4.5. ELISA

The SASP-related indicators in senescent cells were detected using ELISA. The conventional sandwich method and high-sensitivity series enzyme-linked immunosorbent assay (ELISA) kits (Servicebio, China) were used to measure the secretion expression levels of SASP-related indicators IL-6 and TNF-α in the culture supernatants of cervical cancer senescent cells and the control group cells (normal cervical epithelial cells/cervical cancer non-senescent cells). The procedure was as follows: required microplate strips were removed, and the remaining strips were sealed and stored in the dark. Standard, blank, and sample wells were set up in duplicate. Each standard well received 100 μL of diluted standard; blank wells received 100 μL of diluent; and sample wells received 100 μL of sample. After incubation at 37 °C for 90 min, the liquid was discarded, and the wells were washed three times. Then, 100 μL of biotinylated antibody working solution was added, followed by another 37 °C incubation for 60 min. After three additional washes, 100 μL of HRP enzyme conjugate working solution was added and incubated for 30 min at 37 °C. Following a final wash, 100 μL of TMB substrate solution was added, and color was developed at 37 °C for 15 min (until a clear gradient appeared in the standard wells). The reaction was stopped with 50 μL of stop solution. Absorbance was measured at 450 nm within 5 min using a microplate reader, with correction at 630 nm (or 570 nm). Sample concentrations of IL-6 and TNF-α were calculated based on the standard curve.

### 4.6. Cell Scratch Experiment

HeLa cells were seeded at a density of 2 × 10^5^ cells per well in a 6-well plate. When confluence reached 80–90%, a 10 μL sterile pipette tip was used to scratch the plate vertically. The plate was washed three times with PBS to remove adherent cells. The experimental groups were treated with complete medium containing different concentrations of Mg^2+^ for 72 h, whereas the control group was treated with serum-free medium. Photographs of the wound region were taken at 0 and 72 h using an automated fluorescence inverted microscope (DMi8, Leica, Germany) with 100× magnification.

### 4.7. Transwell Invasion Assay

A 1:8 mixture of Matrigel (Corning, Corning, NY, USA) and DMEM was prepared, and 60 μL was spread uniformly onto the upper Transwell chamber (NEST, Wuxi, China) and left to solidify at 37 °C for 3 h. After discarding any unsolidified Matrigel, the gel was rehydrated with 50 μL DMEM. Then, 3 × 10^5^ cells were seeded into the upper chamber, and the lower chamber was filled with 500 μL of complete medium plus 10% FBS. As mentioned above, the upper chamber medium in the experimental group contained different concentrations of Mg^2+^, and the control group was serum-free medium. Cells were cultured for 72 h. Thereafter, the Transwell insert was detached, the medium was removed, and the insert was rinsed twice with PBS. After wiping off the upper-surface cells with a cotton swab, the insert was fixed in 4% paraformaldehyde (20 min), washed twice with PBS, stained with 0.1% crystal violet (15 min), and washed twice again. Images were obtained with a fluorescence inverted microscope (Leica DMi8 automated, Germany) at 200× magnification.

### 4.8. Western Blot

Total protein was extracted from cells with precooled RIPA lysate (Beyotime, China); the supernatant was collected by centrifugation at 12,000× *g* for 15 min at 4 °C. A BCA protein quantification kit (Servicebio, China) was used to determine protein concentration. The protein concentration was adjusted to 2 μg/μL, then 4 × SDS loading buffer was added and denatured with boiling for 10 min. Subsequently, 10 μg protein samples were subjected to 10% SDS-PAGE (220 V, 30 min). Proteins were transferred to a 0.2 μm PVDF membrane (Millipore, Burlington, MA, USA) by wet rotation (400 mA, 25 min). The cells were blocked with protein-free rapid blocking solution (Servicebio, China) for 10 min at room temperature. Primary antibodies (rabbit anti-human) were incubated at 4 °C for 12 h and included p21 (1:1000, AB109520, Abcam, Cambridge, UK), laminB1 (1:1000, ab133741, Abcam, UK), ATM (1:5000, ab32420, Abcam, UK), p-ATM (1:50,000, ab81292, Abcam, UK), CHK2 (1:50,000, ab109413, Abcam, UK), p-CHK2 (1:10,000, ab85743, Abcam, UK), and GAPDH (1:5000,ab181602, Abcam, UK); the PVDF membrane was washed with 1 × TBST (10 min × 3). The HRP-labeled secondary antibody (1:500, Abcam, UK) was incubated at room temperature for 1 h, followed by washing with 1 × TBST (10 min × 3). The membranes were developed by using an ECL chemiluminescence reagent (Biosharp, Beijing, China), and the images of the proteins on the membrane were acquired using a chemiluminescence imager (600, AzureBiosystems, Dublin, CA, USA). ImageJ 1.52v software was used to analyze the gray values of the western blot images. For statistical analysis, the grayscale value of each protein was normalized to that of GAPDH. The relative phosphorylation levels of ATM and CHK2 were then calculated by comparing their phosphorylated forms (p-ATM, p-CHK2) to the corresponding total protein levels, respectively.

### 4.9. Construction of GFP-Labeled HeLa Cells

HeLa cells were added to 6-well plates at a concentration of 1 × 10^6^ cells/mL and kept overnight in a 37 °C incubator containing 5% CO_2_ until the cells became fully attached. The amount of virus needed was calculated according to the optimal multiplicity of infection (MOI) of lentivirus (MOI = 10). The transfection system (containing lentivirus and infection enhancement solution) was prepared by using the complete medium without antibiotics (95% DMEM + 5% FBS) and was added to the well plate for co-culture with cells. At 48 h following transfection, both cell health and fluorescence expression were assessed via fluorescence microscopy. Subsequently, puromycin was applied at a suitable concentration for screening. The screening continued for 7 days, resulting in the death of all non-transfected cells and the survival of only stably transfected cells, thereby demonstrating that the transfection had been successful.

### 4.10. Establishment of Cervical Cancer Xenograft Model

All experiments on animals were approved by the Ethics Committee for Experimental Animal Welfare of Xinjiang Medical University (ethics approval No. IACUC-JT-20250804-01). Female BALB/c nude mice (purchased from Beijing Weitong Lihua Laboratory Animal Technology Co., Ltd., Beijing, China) were reared under standardized conditions: 21–23 °C, 12-h light/dark cycle, and relative humidity of 45–65%. They had unrestricted access to water and food. Subcutaneous tumor xenografts were established in 6-week-old mice (weighing 20–25 g) by injecting 100 μL of GFP-tagged HeLa cells (1 × 10^8^ cells/mL).

A total of 30 mice carrying subcutaneous tumors measuring 5 ± 1 mm in diameter were randomized into five experimental groups, with six mice per group (*n* = 6): MgCl_2_ injection groups (5 mm, 10 mm, 15 mm, 20 mM) and a control group. The control group was injected with normal saline. A 29G microsyringe (Shanghai Yuyan, Shanghai, China) was used for intratumoral multi-point injection (50 μL/time). The needle hole was pressed for 1 min after injection to prevent leakage; the intervention was given once every other day for 20 consecutive days. The feeding and activity of the mice were observed every 5 days, and the body weight of the mice was measured. The short and long diameters of the tumors were measured, and the tumor volume was calculated using V = (length × width^2^)/2. The weight change and tumor volume curves for the mice were then drawn.

### 4.11. Small Animal In Vivo Imaging Analysis

A total of 24 h after the last administration, mice were anesthetized by intraperitoneal injection of 1% sodium pentobarbital and placed in the small animal in vivo imaging system. Transfer images were taken with an excitation light wave of 465 nm and a received light wave of 760 nm.

### 4.12. Histopathological Evaluation

After euthanasia, an incision was made through the skin, and the tumor tissue was fully excised. After being weighed, the slices were cut along the maximum diameter line; one half was immediately frozen in liquid nitrogen, and the other half was fixed in 4% paraformaldehyde for 48 h. The cells were dehydrated with gradient ethanol, dipped in wax, embedded in paraffin, and stained with H&E after serial sections at 4 μm. Images were taken using a microscope (OLYMPUS, U-AW, Tokyo, Japan), with magnifications were 100× and 400×, respectively. Then the images were read by two senior pathologists in a double-blind manner.

### 4.13. Blood Biochemical Test

Following collection, blood samples were mixed for 10 min and then centrifuged at 3000 rpm for about 20 min at 4 °C to obtain the supernatant. An automated analyzer (Hitachi, 7600-030, Tokyo, Japan) was used for blood biochemical measurements, including alanine aminotransferase (ALT), aspartate aminotransferase (AST), and alkaline phosphatase (ALP) to assess liver function and UREA to assess renal function.

### 4.14. Detection of Serum Magnesium Concentration

Blood samples were collected and after being mixed for 10 min, the supernatant was collected by centrifugation at 3000 rpm for about 20 min at 4 °C. Serum magnesium concentration was measured by methyl thymol colorimetry.

### 4.15. Statistical Analysis

A minimum of three replicates was used for all in vitro and in vivo studies. Values are denoted as mean ± SD. Statistical analysis and graphing of all data were performed using GraphPad Prism 9.0 software. Differences between groups were analyzed using one-way analysis of variance (ANOVA). *p* < 0.05 was considered statistically significant.

## 5. Conclusions

These findings should be interpreted within the context of a preclinical, high-dose local Mg^2+^ model. Within this context, this study systematically confirmed that high concentrations of magnesium ions can selectively inhibit the proliferation, migration, and invasion of HeLa cells, ultimately inducing a senescence-like state by activating the classic DNA damage response pathway, specifically the ATM/CHK2/p21 signaling axis. The use of an ATM inhibitor confirmed the functional role of this pathway in mediating the observed upregulation of p21. The anti-tumor efficacy and favorable biosafety of this local application strategy were further verified. In vivo experiments further verified the anti-tumor efficacy and good biosafety of this local application strategy, without causing any systemic toxic side effects. Collectively, these findings provide new molecular evidence for the anti-cancer mechanism of magnesium and establish a solid experimental and theoretical basis for developing safe, effective, and low-cost local treatment strategies, such as postoperative adjuvant therapy using degradable magnesium materials. These findings not only provide new molecular evidence for the anti-cancer mechanism of magnesium, but also provide a solid experimental and theoretical basis for the development of safe, effective, and low-cost new strategies for local treatment of cervical cancer, such as postoperative adjuvant therapy based on degradable magnesium materials.

## Data Availability

The original contributions presented in this study are included in the article. Further inquiries can be directed to the corresponding author.
